# Genetic Variants of Interleukin-4 in Romanian Patients with Idiopathic Nephrotic Syndrome

**DOI:** 10.3390/medicina58020265

**Published:** 2022-02-10

**Authors:** Ioana Tieranu, Cristian George Tieranu, Monica Irina Dutescu, Camelia Elena Berghea, Mihaela Balgradean, Olivia Mihaela Popa

**Affiliations:** 1 Department of Pediatrics, “Carol Davila” University of Medicine and Pharmacy, Dionisie Lupu Street No. 37, Sector 2, 020021 Bucharest, Romania; ioana.tieranu1@drd.umfcd.ro (I.T.); camelia.berghea@umfcd.ro (C.E.B.); mihaela.balgradean@umfcd.ro (M.B.); 2 Department of Pediatrics, Clinical Emergency Hospital for Children ‘Marie Sklodowska Curie’, Constantin Brancoveanu Bulevard No. 20, Sector 4, 077120 Bucharest, Romania; 3 Department of Gastroenterology, “Carol Davila” University of Medicine and Pharmacy, Dionisie Lupu Street No. 37, Sector 2, 020021 Bucharest, Romania; 4 Department of Hematology, “Prof. Dr. Constantin T. Nicolau” National Institute of Blood Transfusion, Constantin Caracas Street No. 2-8, 011155 Bucharest, Romania; monica_dutescu@yahoo.com; 5 Department of Pathophysiology and Immunology, “Carol Davila” University of Medicine and Pharmacy, Dionisie Lupu Street No. 37, Sector 2, 020021 Bucharest, Romania; olivia.popa@umfcd.ro

**Keywords:** idiopathic nephrotic syndrome, cytokines, interleukin-4, single-nucleotide polymorphisms, corticosteroids

## Abstract

*Background and objectives*: One of the most frequent glomerular diseases in the pediatric population is represented by the idiopathic nephrotic syndrome (INS). The exact mechanisms mediating the disease are still unknown, but several genetic factors have been studied for possible implications. Cytokines are considered to play a pivotal role in mediating INS disease progression, interleukin-4 (IL-4) exhibiting particular interest. The objective of this research project was to investigate the association between two *IL-4* gene single-nucleotide polymorphisms (SNPs) and INS susceptibility as well as response to steroid therapy, in a group of Romanian children. *Materials and Methods*: In total, 75 patients with INS and 160 healthy controls of Romanian origin were genotyped for *IL-4* rs2243250/−590C/T and rs2070874/−34C/T using real-time polymerase chain reaction. Association tests were performed using the DeFinetti program and Plink 1.07 software and *p*-values < 0.05 were considered statistically significant. *Results*: The analysis of INS patients and controls revealed a similar genotype distribution of the studied SNPs. The minor T alleles were less frequent in the INS group, but not statistically significant (*p* = 0.1, OR = 0.68 and *p* = 0.2, OR = 0.74). Regarding the response to steroids, a low frequency of 590*T allele in steroid-resistant patients (7.7%), compared with steroid-sensitive patients (14%) and controls (17.5%), was obtained, but the difference did not reach the statistical significance threshold. The same result was obtained for −34C/T SNP. *Conclusions*: This is the first study examining the relationship between the *IL-4* gene and INS susceptibility conducted in a European population, and particularly in Romania. The investigated SNPs were found to not be associated with disease susceptibility or response to the steroid treatment of pediatric INS.

## 1. Introduction

Nephrotic syndrome (NS) is a clinical renal condition characterized by proteinuria, hypoalbuminemia, edema and hypercholesterolemia [1]. About 90% of cases of NS are idiopathic (INS), the remaining 10% being secondary to systemic pathologies such as infections (HIV, toxoplasmosis and congenital syphilis), malignancies (leukemia and lymphoma) and immunological diseases (Castleman’s disease) [2,3]. Depending on the etiology, NS may lead to a gradual loss of kidney function, followed by the necessity of dialysis or kidney transplant [4]. Despite several advances in INS pathophysiology, its clinical evolution is still unpredictable [5].

INS is the most frequent glomerular disorder of childhood, having a variable incidence (between 1.99 and 16.9 per 100,000 children) depending upon the patient’s ethnicity or country of origin [6,7]. It can be classified according to a number of parameters, although the most common criteria aim to dichotomize the disease based on the clinical response to corticosteroid treatment into steroid-sensitive (SSNS) or steroid-resistant (SRNS) [8]. However, despite being helpful in guiding therapy, this classification does not offer answers to the questions regarding the disease mechanisms [9].

Based on pathology reports from renal biopsies, the majority (85%) of INS patients have minimal change NS (MCNS), with the remaining cases exhibiting other histopathological types [2].

Although the pathophysiology of the disease is highly complex and remains largely unknown, several studies have suggested that INS is a primary immune-mediated disorder driven by an altered balance between T helper 1 (Th1) and T helper 2 (Th2) cytokines [10]. The efficacy of immunosuppressive treatments that focus on inhibiting T cell functions is high. For example, chemotherapy treatments used in targeting T cell populations in T cell lymphomas (i.e., Hodgkin’s lymphoma that can trigger or precede NS) are able to mediate disease control and represent supportive evidence that dysfunctions/dysregulations of T lymphocytes play an important role in this pediatric disease [4,11,12].

The upregulation of specific Th2 cytokines (IL-4, IL-5, IL-9, IL-10 and IL-13) has previously been shown to promote the development of NS [13]. Moreover, recent data point to the fact that cytokines play a major role as mediators of inflammation, some of them being considered prime candidates for mediating INS progression [14,15,16].

Apart from the involvement of T cells, an important role of B cells in the pathogenesis of INS has emerged through the therapeutic efficacy of Rituximab—an anti-CD20 monoclonal antibody which maintains a prolonged remission that persists in most cases at least until B cells recover [17]. Furthermore, elevated circulating levels of total B cells have already been identified in steroid-sensitive pediatric patients at disease onset [18], with data showing that memory B cells, more than other B cell subsets, are increased in this group [19]. Despite the accumulating evidence, the role of B cells in the pathogenesis of INS is still unknown, and the mechanism responsible for the effectiveness of Rituximab remains to be identified.

Interleukin-4 (IL-4) is an immunoregulatory cytokine secreted by basophils, mast cells and T cells. It was initially identified as a co-mitogen of B-cells, but was subsequently shown to be involved in conditions such as tissue repair and homeostasis through macrophage activation, immunoglobulin E class switching in B cells and Th2-cell-mediated immunity [20,21,22,23,24]. Several early studies on macrophages showed an anti-inflammatory activity of IL-4, when administered as a response to an inflammatory stimulus, being able to downregulate the production of the cytokine line TNF [25]. However, IL-4 is not totally an anti-inflammatory agent: in vivo studies correlated chronic high dosage and/or overproduction of IL-4 with weight loss, decreased pro-inflammatory cytokine production, histiocytosis and increased IFN-gamma production [26].

The *IL-4* gene is part of the Th2 cytokine gene cluster on the human chromosome 5q23-31 together with *IL-13* and *IL-5* genes [27]. The promoter of the *IL-4* gene contains a regulatory polymorphism, rs2243250 (−590C/T), that affects the binding of the nuclear factor for activated T cells (NFAT), one of the main transcriptional activators of *IL-4* in T cells. In vitro and in vivo studies show that the presence of the minor allele T contributes to the appearance of a supplementary NFAT binding site, which will affect the transcription rate (a threefold greater expression than the −590C allele) [28]. A more recent study, which included patients of north European heritage, reported the influence of both −590C/T and −34C/T promoter polymorphisms on *IL-4* gene expression and IL-4 secretion. The presence of −590TT and −34TT genotypes was associated with increased expression and secretion compared with −590CC and −34CC genotypes [29].

Recent studies have shown an association between −590C/T polymorphism and the risk of MCNS in the Asian population [30,31]. However, in the European population, studies failed to show associations of single nucleotide polymorphisms (SNPs) of this Th2 cytokine with the susceptibility to this histological INS pattern [15,32].

Regarding the steroid resistance in INS patients, despite extensive research in the field, no early and reliable predictor of steroid treatment response has been found for translation into clinical practice. Moreover, a literature review of different gene variations related to steroid therapy response resulted in discordant conclusions. Not only cytokine genes, but also steroid-metabolism-related genes, displayed contradictory results in various ethnic groups [33,34].

The present study aimed to identify a possible association between *IL-4* gene polymorphisms and the risk of INS in a group of Romanian children. We also studied the influence of these SNPs on the response to steroid treatment.

## 2. Materials and Methods

### 2.1. Patients and Controls

A total of 235 unrelated Caucasian individuals of Romanian origin were included in the study. The Romanian ethnicity was certified based on the surname of the subjects accompanied by a self-reported statement that at least three ancestral generations had lived within Romanian territory. INS patients (N = 75) were recruited from the Nephrology Department of the “M.S. Curie” Emergency Hospital for Children, Bucharest, Romania. All patients with INS were diagnosed according to the Kidney Disease Improving Global Outcomes (KDIGO) guideline criteria and were stratified according to their response to corticosteroid therapy: steroid-sensitive, the attainment of complete remission within initial 4 weeks of corticosteroid therapy; and steroid-resistant, a failure to achieve complete remission after 8 weeks of corticosteroid therapy [8].

Inclusion criteria were aged between 12 months and 18 years old, certified Romanian heritage and an established diagnosis of INS based on the above-mentioned recommendations.

Exclusion criteria were infantile congenital and secondary nephrotic syndromes.

The control group consisted of 160 healthy control subjects, with no history of proteinuria and/or edema.

### 2.2. Ethical Considerations

The study was approved by the Local Ethics Committee of “M.S. Curie” Hospital, Bucharest, Romania, with registration number 16266/10.06.2015. The details were explained to all parents and controls and consent for genetic screening was obtained. The study was performed in accordance with the principles stated in the Declaration of Helsinki and its later revisions.

### 2.3. DNA Extraction and Genotyping

Genomic DNA was obtained from EDTA-treated peripheral blood samples using the QIAamp DNA Blood Mini Kit (Qiagen, Hilden, Germany) according to the manufacturer’s protocol.

Two single nucleotide polymorphisms of *IL-4* gene were selected for this study: rs2243250 (−590C/T) and rs2070874 (−34C/T). The SNP genotyping was performed by real-time polymerase chain reaction with TaqMan Allelic Discrimination Assays C_16176215_10 and C_16176216_10, respectively (Real-time PCR System, Applied Biosystems by Thermo Fisher Scientific, Foster City, CA, USA).

### 2.4. Statistics

Mann–Whitney U tests were used to compare the variables with non-parametric distribution between groups. For categorical variables the chi-squared test was applied. Statistical analysis was performed using the Statistical Package for Social Science (SPSS v17.0).

The Hardy–Weinberg equilibrium (HWE) was verified using the chi-squared test. Association tests and HWE tests were performed with the DeFinetti program; *p*-values ≤0.05 were considered statistically significant [35].

Alleles and genotype frequencies of the studied SNPs were compared between INS patients and controls, and between the steroid-sensitive group and the steroid-resistant group.

The estimations of haplotype frequencies and association tests were performed using Plink 1.07 software (https://zzz.bwh.harvard.edu/plink/, accessed on 1 December 2021). Genotype data from controls were used to estimate intermarker linkage disequilibrium (LD) by calculating pairwise r^2^ with Plink 1.07.

## 3. Results

A total of 75 patients diagnosed with INS and 160 sex-matched healthy controls participated in the study. Demographic characteristics of the subjects are presented in Table 1. The controls and patients’ groups showed no departure from HWE for all studied SNPs. The genotyping success rate was 100%.

The data for the control group were compared with data available in the NCBI Database of Short Genetic Variations (dbSNP) for samples of European descent. The frequencies of alleles and genotypes for both SNPs were similar in our control group compared with the published data for European population (HapMap-CEU).

The frequencies of *IL-4* gene SNP genotypes and alleles in control and INS patients are presented in Table 2. The minor T alleles were less frequent in the INS group than the control group for both investigated polymorphisms, but not statistically significantly. The analysis of the two groups revealed a similar genotype distribution of the studied SNPs (Table 2).

The r^2^ value showed a medium level of intragenic LD in our population (r^2^ = 0.72). Two haplotypic variants had frequencies over 5% in controls: rs2243250/rs2070874 CC, 81%, and TT, 16%. We found no association of *IL-4* haplotypes with the risk of INS.

INS patients were subdivided based on steroid treatment responsiveness. Although minor alleles were under-represented in steroid-resistant patients, no significant statistical difference in allele and genotypes frequencies was found between the steroid-sensitive and steroid-resistant subgroups when compared with controls (Table 3), nor when compared with each other (data not shown).

## 4. Discussion

The aim of our study was to analyze the association between two *IL-4* gene polymorphisms (−590C/T and −34C/T) and the susceptibility to INS in a group of pediatric Romanian patients. We also investigated if any of these polymorphisms correlated with the response to corticosteroid therapy. To the best of our knowledge, this is one of the few published studies addressing the relationship between *IL-4* gene and INS susceptibility conducted in a European population, and the first to focus in a Romanian population.

IL-4 is a key immunomodulatory cytokine and is involved in the differentiation of B lymphocytes through regulation of the IgG4 isotype switch to IgE. It produces an increased expression of major histocompatibility complex class II (MHC II), inhibits the development of Th1 cells, and also stimulates Th2 differentiation from Th0 cells, promoting their growth and proliferation [36].

Measurements of cytokine levels, such as IL-4 and IL-13, in the serum or urine of INS patients has been shown to be increased in those who experienced relapse in disease evolution [14,37]. T helper 2 (Th2) cells are defined by the production of the collectively called type 2 cytokines: IL-4, IL-5 and IL-13 [38]. These results depict INS as a Th2-dependent glomerular disease. This hypothesis is further sustained by the association of INS and allergic disorders such as allergic rhinitis, asthma, and eczema that are typically associated with a Th2 immunologic response. However, although the role of type 2 cytokine genes is well documented for allergic diseases [39,40,41], only a few studies have been published for INS, and these involved mainly Asian populations [30,31,42].

Two studies, reported by Tripathy et al. [43] and Jafar et al. [29], were conducted on the same group of 150 INS patients from India for several cytokine gene polymorphisms. For *IL-4* −590C/T polymorphism, the comparison of INS patients with 569 controls showed the association with disease risk (C vs. T: *p* = 0.04; OR = 1.38). When the steroid-resistant group (N = 35) was compared with the steroid-responsive group (N = 115), association was found at the genotypic level (CC vs. TT: *p* = 0.020; OR = 6.46) [42].

The results of the studies cited above are in contrast with those we obtained and with those reported by Madani et al. in 2014 [44]. The latter was a case–control study that included 60 INS pediatric patients and 30 controls from Egypt. The authors investigated the implications of several cytokine genes promoter polymorphisms (*IL-6* -G174C, *IL-4* −C590T and *TNF-alpha* -G308A) in the corticosteroid treatment response. Their results did not show any significant differences between the patients and the control groups, nor between the steroid-sensitive and the steroid-resistant subjects, regarding the *IL-4* −C590T genotypes or allele distributions. Interestingly, a significant association was obtained regarding the *TNF-alpha* −308G/A SNP, with the AA genotype and the minor A allele being more frequent in the patient group—in contrast with our previously published data [45]. The latest data regarding *IL-4* −C590T SNP and INS was published in 2020, showing no association between this polymorphism and the predisposition to the disease in Kuwaiti patients [46].

Similar results for *IL-4* −590C/T SNP were reported by Malik et al. [47] from a sample of Iraqi patients showing no significant difference between the frequencies of the minor T allele when comparing the steroid-sensitive group with the steroid-resistant group. Even though more than half of the steroid-sensitive patients have the heterozygous genotype (CT) of this polymorphism—compared with only 30% of the steroid-resistant patients—this difference failed to reach the statistical significance threshold.

Several studies have correlated genetic variations of *IL-4* gene and predisposition to a subtype of nephrotic syndrome—minimal change nephrotic syndrome (MCNS)—and consider these variants predictors for frequently relapsing childhood SSNS [30,31,48]. Another investigation of the role of IL-4 in the pathogenesis of MCNS found significantly increased levels of *IL-4* mRNAs both in mitogen-stimulated and unstimulated MCNS peripheral blood lymphocytes compared with healthy controls [14].

Interesting results were published by Kobayashi et al. [30] from a study that included 58 Japanese pediatric patients with MCNS and 63 controls. The frequencies of the −590T allele and of the TT genotype were significantly lower in INS patients than controls. In contrast to these data, an Indonesian study revealed significant differential genotypic distribution of the *IL-4* −C590T polymorphism between pediatric patients with MCNS and controls (*p* = 0.02). The prevalence of CC homozygotes was significantly lower in the MCNS patients than in the controls, with the frequency of C allele also being significantly lower in the first group (*p* = 0.0006) [31].

Regarding the −C590T *IL-4* single nucleotide polymorphism, the data discussed above are abundant and widely variable as results; to the best of our knowledge, this is the first study to investigate the −34C/T *IL-4* gene polymorphism for its role in INS disease susceptibility and treatment response. No significant differences were observed between INS group and controls for genotype and allele frequencies, nor regarding the relationship with response to corticosteroid treatment.

An important limitation of the present study is represented by the small sample size included in the final analysis, with a low number of SRNS patients. Further research on a larger scale is necessary to confirm these results. The lack of data from other European populations makes it very difficult to draw a clear conclusion regarding IL-4 involvement in the pediatric INS development or response to corticosteroid treatment.

## 5. Conclusions

In conclusion, neither −590C/T, nor −34C/T polymorphisms of *IL-4* gene are associated with the susceptibility for developing INS, or with the response to corticosteroid treatment, in the Romanian population. The discrepancy between our results and previously reported findings may be due to the small number of patients available in the diseased cohort, especially steroid-resistant subjects. Additionally, in our opinion, the genetic background of different study populations, as well as a complex genetic determination for resistance to treatment, can be incriminated.

## Figures and Tables

**Table 1:** Demographical features of the patients enrolled in the study.

	INS Group (*n* = 75)	Control Group (*n* = 160)	*p*-Value
Age (Mean ± SD)	4.74 ± 3.29	37.73 ± 13.72	*p* < 0.0001
Gender (%)			
Male	73.3%	65.6%	*p* = 0.237
Female	26.7%	34.4%	
Response to steroids (%)			
SSNS	82.6%	N/A	
SRNS	17.4%		

**Table 2:** Genotypes and allele frequencies of *IL-4* gene polymorphisms among INS and control groups.

SNP	INS (N = 75)	Controls	* *p*-Value OR	95% CI
***IL-4*** −**590C/T** **rs2243250**				
Genotypes				
CC	57 (76%)	107 (67%)		
CT + TT	17 + 1 (24%)	50 + 3 (33%)	*p* = 0.15	0.63 (0.34–1.19)
Minor allele				
T	19 (12.6%)	56 (17.5%)	*p* = 0.18	0.68 (0.39–1.19)
***IL-4*** −**34C/T** **rs2070874**				
Genotypes				
CC	56 (75%)	109 (68%)		
CT + TT	18 + 1 (25%)	47 + 4 (32%)	*p* = 0.30	0.72 (0.39–1.34)
Minor allele				
T	20 (13.3%)	55 (17%)	*p* = 0.28	0.74 (0.42–1.28)

* *p*-value < 0.05 is significant; OR, odds ratio; CI, 95% confidence interval; INS, idiopathic nephrotic syndrome.

**Table 3:** Genotypes and allele frequencies of *IL-4* gene polymorphisms among SSNS, SRNS and control groups.

SNP	Controls (N = 160)	SSNS (N = 62)	Statistics	SRNS (N = 13)	Statistics
*IL-4* −590C/T rs2243250					
Genotypes					
CC	107 (67%)	46 (74%)		11 (85%)	
CT + TT	50 + 3 (33%)	15 + 1 (26%)	*p* = 0.29 OR = 0.70 95% CI 0.36–1.35	2 + 0 (15%)	*p* = 0.18 OR = 0.36 95% CI 0.07–1.71
Minor allele T	56 (17.5%)	17 (14%)	*p* = 0.33 OR = 0.74 95% CI 0.41–1.34	2 (7.7%)	*p* = 0.2 OR = 0.39 95% CI 0.09–1.71
*IL-4* −34C/T rs2070874					
Genotypes					
CC	109 (68%)	45 (73%)		11 (85%)	
CT + TT	47 + 4 (32%)	16 + 1 (27%)	*p* = 0.51 OR = 0.80 95% CI 0.42–1.54	2 + 0 (15%)	*p* = 0.21 OR = 0.38 95% CI 0.08–1.81
Minor allele T	55 (17%)	18 (14.5%)	*p* = 0.49 OR = 0.81 95% CI 0.45–1.45	2 (7.7%)	*p* = 0.27 OR = 0.40 95% CI 0.09–1.74

OR, odds ratio; CI, 95% confidence interval; SSNS, steroid-sensitive nephrotic syndrome; SRNS, steroid-resistant nephrotic syndrome.

## Data Availability

Raw data for the final analysis is available at the corresponding author upon reasonable request.
